# Factors associated with COVID-19 vaccination in Belize

**DOI:** 10.1016/j.jvacx.2023.100380

**Published:** 2023-09-01

**Authors:** Diego Rios-Zertuche, Giuliana Daga, Filippo Iorillo, Ana Mylena Aguilar Rivera, Melissa Diaz-Musa, Natalia Largaespada Beer, Florencia López Boo, Julio Sabido

**Affiliations:** a Salud Mesoamerica Initiative, Inter-American Development Bank, USA; b Inter-American Development Bank, Washington, DC, USA; c MSc Economics, Consultant, Portugal; d Inter-American Development Bank, Dominican Republic; e Ministry of Health and Wellness, Belize

**Keywords:** Belize, COVID-19 vaccines, COVID-19 pandemic, Vaccine uptake, Vaccine hesitancy, Vaccination intentions

## Abstract

**Background:**

We analyzed the factors associated with vaccine uptake, vaccination intention, and reasons for vaccine hesitancy in Belize.

**Methods:**

We collected a nationally representative household survey of the population in Belize aged 15 years and older (September—October 2021). We compared potential correlates between vaccinated and unvaccinated people using Chi-square tests. Then, we performed logistic regression analysis to identify factors associated with vaccination uptake among all respondents and vaccination intention among the unvaccinated.

**Results:**

Our analysis included data from 1261 interviews. Nearly four out of every five people reported being vaccinated, having received at least one dose. Adolescents and young adults were more likely to be unvaccinated. Significant differences were observed for most behavioral variables. Among the 41.7% of the unvaccinated respondents who said they would probably not or definitely not get vaccinated, the primary reason for their hesitation was fear of side effects. Additionally, almost one third of the unvaccinated individuals expressed a lack of trust in vaccines. Factors associated with increased likelihood of vaccination were efficacy beliefs, self-efficacy and vaccine attitudes. People who believed it was easy to get a vaccine were over 23 times (OR 23.63 [95% CI: 14.21–39.27]) more likely to be vaccinated, while those who believed in vaccine safety were 2.57 times [OR 95% CI: 1.52–4.35] more likely to be vaccinated. Among the unvaccinated, factors associated with intention to get vaccinated were self-identifying as Garifuna and having clear efficacy beliefs.

**Conclusions:**

To our knowledge, this is the first study describing factors associated with COVID-19 vaccine uptake and vaccine hesitancy in Belize. Our findings revealed that accessibility has been the primary limitation in increasing vaccine coverage, and <7% of the eligible population have been strong vaccine deniers. To enhance vaccine uptake, targeted outreach efforts are necessary to address access barriers. Our results call for increased efforts improving self-efficacy, efficacy beliefs, and perceived norms.

## Highlights

• Accessibility was the largest limitation to increase vaccine coverage in Belize.
• Vaccine attitudes had an important role in vaccine uptake.
• Only a small proportion of the population (6.6%) were strong vaccine deniers.
• Vaccine uptake among adolescents and young adults was lagging.
• Fear of side effects limited vaccine uptake in people with preexisting conditions.

## Introduction

By August 2022, over 200 countries had started vaccinating against COVID-19 [1]. However, while over one third of the global population had received at least one vaccine dose, the same was true for only about one fifth of people living in low-income countries. Among the countries in Latin America, Cuba had the highest proportion of people vaccinated against COVID-19 with at least one dose [2], while Haiti the lowest, with <3 percent.

To achieve herd immunity against SARS-CoV-2, vaccine coverage was estimated to be between 60% and 90% of the total population [3]. In Belize, at least 493,758 doses had been administered, covering about 63.2% of the population [1], or roughly 124 doses per 100 people [2]. Four vaccines had been approved and delivered since January 2021, namely Johnson & Johnson, Oxford/AstraZeneca, Pfizer/BioNTech, and Sinopharm/Beijing. Despite significant evidence of the value of vaccines in preventing symptomatic and severe illness, vaccine hesitancy has been a growing concern as efforts intensified to increase vaccine uptake.

Research has shown that failing to achieve the targeted level of herd immunity can lead to virus mutations that increase their virulence and transmissibility. An example of this phenomenon was observed in India, where low vaccination rates directly correlated with a rise in mortality [4]. Therefore, it is crucial to prioritize the rapid expansion of vaccine distribution and address any existing vaccine hesitancy to effectively combat the spread of the virus.

Studies have found different factors influencing COVID-19 vaccine uptake as well as reasons behind vaccine hesitancy. Existing literature on vaccination intentions and actions, combined with new insights from the COVID-19 pandemic, highlight the significant role of behavioral barriers could play in reducing vaccine hesitancy. For instance, Dai et al. [5] demonstrated that low-cost SMS reminders in the United States significantly increased the probability of vaccination. Results suggest that psychological barriers hindered vaccination even among individuals who had already decided to get vaccinated. Additionally, their research revealed that a campaign aimed at addressing misperceptions and beliefs on vaccination was less successful. This suggests that influencing the decision to get vaccinated likely requires a nuanced and thorough approach. Social and behavioral sciences can play a pivotal role in effectively targeting messages and interventions to promote vaccine uptake [6].

Existing evidence suggests that drivers of vaccine uptake and hesitancy vary across different contexts. In wealthier countries, vaccine hesitancy tends to be more common among young people, women, people with lower income or lower education, those with limited COVID-19 exposure, reduced trust in government, residents of disadvantaged, areas and people adhering to right-wing political views [7]. A study conducted in Belgium identified several factors associated with vaccinations, including higher age, positive government opinion, increased medical risk, language spoken (Dutch vs. French) and knowing someone who was hospitalized [7]. Hesitancy was found to be higher among people below 54 years old [7]. In the United States [8], higher probable vaccine efficacy was linked to increase vaccine uptake, while minor side effects did not significantly affect uptake. Other factors associated with higher vaccine uptake include engagement in community activities, trust in physicians and family doctors, and trust in government health authorities [9], [10]. Interestingly, financial incentives did not seem to increase uptake among the vaccine hesitant [11]. In Chile, several factors were found to decrease vaccines uptake, including decreased fear of contagion, family opinions, misinformation, health union recommendations, and perceived risk [12]. Further, individuals tended to prefer vaccines with fewer side effects over those with higher effectiveness [12]. Moreover, pregnant and breastfeeding women with lower education and/or unemployed were less willing to get vaccinated [13], [14].

While there is already a wealth of evidence of interventions to reduce intention actions gaps in vaccine uptake [15], such evidence is relatively scarce for COVID-19 vaccines and often focuses on high-income countries [5], [16], [17], [18]. This discrepancy is particularly concerning in low-income and middle-income countries, which not only face challenges in accessing vaccines but may also be disproportionately affected by lower trust in scientists and compliance with health-related policies [19]. Moreover, some of the psychological barriers that prevent individuals to take COVID-19 vaccines are not directly related to the intention-action gaps [5] but are rooted in perceptions and beliefs concerning vaccines and science in general. Therefore, it is important to understand the specific barriers in the context of low-income countries.

In Belize, the vaccination campaign was primarily led by the Ministry of Health and Wellness (MOHW). Initially, the focus was on vaccinating high-risk population groups according to priority categories. As the vaccine supplies increased, the campaign expanded its coverage to include all adults in the country, with vaccines provided free of charge to all eligible individuals. Beginning in February 2022, Belize extended its vaccination campaign to include individuals between the ages of 12 and 17 years. Later, in May 2022, the age range was further expanded to encompass children between 5 and 12 years.

In this study, we applied the Integrative Model of Behavioral Prediction [20] to analyze the factors associated with vaccine uptake and vaccination intention in Belize. The model establishes that behavior is mainly determined by intentions. Intentions, in turn, are a function of attitudes, in other words, personal favorable or unfavorable views of the behavior; perceived norms, or perceptions about others approval or disapproval of the behavior; and self-efficacy, which is the person’s own ability to perform the behavior [21]. Skills and the environment are moderators of the relationship between intentions and behavior. We seek to provide information to strengthen the design of behavior-change interventions aimed at increasing the vaccine uptake and reducing vaccine hesitancy in the country.

## Methods

### Sample selection

We collected national household sample data representative of the population in Belize aged 15 years and older. The sample was obtained through a two-stage sampling process. First, a list of census areas in Belize was obtained using the Belize Population & Housing Census, 2010. In each district, a sample of census areas was randomly selected with probability proportional to size. When a census area had <150 people, nearby census areas were also selected. Six areas were selected in the two most populated districts, and five in all others. Then, we used systematic sampling to select approximately 45 households in each community.

### Instruments

Instruments were designed to obtain a rapid assessment of coverage of maternal and child health interventions, assess impact of the COVID-19 pandemic on health, and evaluate strategies to improve the COVID-19 vaccination roll-out. The questionnaire was organized depending on household members and survey informants. After obtaining informed consent, the initial informant was asked about the usual household members and household characteristics. If the initial informant was a woman in reproductive age or caretaker of children under 5 years old, the interview continued with reproductive, maternal and child health sections. In any other case, these sections were skipped and, after completing the initial interview, an additional informant was asked to answer the maternal and childcare sections separately. In any case, the initial informant was asked about knowledge, aptitudes, and practices related to COVID-19 and COVID-19 vaccinations.

The majority of the questionnaire was adapted from instruments used previously in Belize [22]. Questions regarding COVID-19 and COVID-19 vaccines were obtained from question banks compiled by the World Health Organization [23] and the Centers for Disease Control and Prevention [24]. Instruments were available in English and Spanish. For specific communities with a high proportion of indigenous people, interpreters were available.

### Electronic data entry

Interviewers collected data using computer assisted personal interviewing (CAPI) software. CAPI was programmed using SurveyCTO (https://www.surveycto.com/) and installed in smartphones, which were used for data entry. The software automatically checked for inter-question response consistency, data entry ranges and followed predefined skip-patterns depending on answers. Data was transferred periodically to a secure server. Data quality was consistently monitored remotely by investigators.

### Data collection, training, and pilot test

Data were collected by four teams of two interviewers between September—October 2021. Interviewers were locally recruited from each district. Interviews lasted between 15 and 30 min, depending on the number of household members and survey respondents. Training took place in two 3-day sessions including a description of the study, data collection protocols and procedures, a full review of the questionnaire, and role-playing. Field training sessions and instrument pilot test took place after completing the training period for two additional days. Fieldwork and data collection procedures included multilayered mitigation measures to reduce the risk of COVID-19 infection for interviewers and community members.

### Ethics and confidentiality

The study was reviewed and approved by the Institutional Review Board of the Ministry of Health and Wellness in Belize. Verbal informed consent was obtained from each study participant. Participation was elective and participants could withdraw at any time. Data were automatically de-identified after each interview was completed.

### Study variables

People who reported being vaccinated against COVID-19 were considered vaccinated, regardless of the number of doses, vaccine type or availability of vaccination record. To measure vaccination intentions, informants were asked about their likelihood of getting vaccinated (see Table 2). As a proxy for environmental constraints, informants were asked if they had been offered a COVID-19 vaccine. To measure vaccination attitudes, we included perceptions of vaccine safety, perceived protection, and vaccine concerns. For self-efficacy, we considered the perceived ease to get vaccinated. To measure perceived norms, we incorporated the perceived support of others to get vaccinated (friends, family, community, and healthcare workers). Similarly, for normative beliefs and motivation, informants were asked about the expectation of others getting vaccinated (friends, family, and community).

Regarding respondents' behavioral beliefs (the belief that a behavior will produce a good or bad outcome), we considered the perceived risk to themselves and others (friends, family members, coworkers) of infection, quarantine, infecting someone else, hospitalization, and death. Finally, for efficacy beliefs, we asked about motivations for vaccination, including protecting themselves and others (family, friends, coworkers, community), returning to or finding work or going back to school, resuming social activities, travelling, and encouraged by others. Distal variables included age (in MeSH categories), sex, education, ethnicity, health conditions, and district. We also asked unvaccinated people for their reasons for being unwilling to get vaccinated. We computed income quintiles using The Equity Tool [25], which we summarized it as a binary variable representing the two lower income quintiles for statistical analysis.

### Statistical analysis

We conducted a comparison of potential correlates between vaccinated and unvaccinated individuals using Chi-square tests for categorical variables. Our analysis was limited to responses with complete data. Then, we computed two logistic regression models: the first aimed to identify factors associated with vaccination uptake among all respondents, and the second focused on vaccination intention within the subsample of unvaccinated individuals.

Both models consider the same dependent variables, with a couple of exceptions: the first model did not take into account intention or environmental constraints, as these factors were already manifested in vaccinated individuals. In contrast, the second model incorporated environmental constraints to assess whether accessibility played a significant role as a barrier to vaccination.

Selection of behavioral variables for the final models was based on the statistical significance of bivariate analyses (results of full models are included in Appendix Table A1). Consistent with recommendations, we conducted separate analyses restricting the sample for male and female respondents [26] for the first model. We used Stata SE 17 for the analyses. We set a significance threshold of p = 0.05 a priori. Furthermore, we assessed the goodness-of-fit using the Hosmer and Lemeshow test [27] for the full model and performed link tests for model specification [28].

## Results

We analyzed data from 1261 interviews (86.9% of total interviews). Almost four out of every five individuals reported being vaccinated at the time of the survey (see Table 1). When examining the differences in demographic characteristics between the vaccinated and unvaccinated groups, we observed that a higher proportion of adolescents and young adults were unvaccinated, while a higher proportion of middle-aged and aged people were vaccinated. Moreover, vaccination status appeared to be associated with income and education levels. A higher proportion of unvaccinated individuals had primary education and belonged to the two lowest income quintiles.

**Table 1:** Demographic characteristics of vaccinated and unvaccinated people in Belize, Unweighted (September–October 2021).

Demographic characteristics	All		Vaccinated		Unvaccinated		p-value
	%	95% CI	%	95% CI	%	95% CI	(Chi–square1)
Have you been vaccinated against COVID–19?							
Yes	79.3%	(77.0–81.5%)					
Gender							0.56
Male	32.8%	(30.3–35.4%)	32.4%	(29.7–35.3%)	34.3%	(28.9–40.2%)	
Female	67.2%	(64.6–69.7%)	67.6%	(64.7–70.3%)	65.7%	(59.8–71.1%)	
Age							0.06
Adolescents and Young adults (15–24)	12.5%	(10.1–15.4%)	11.3%	(9.3–13.7%)	17.1%	(11.3–25.1%)	
Adults (25–44)	44.1%	(40.0–48.2%)	44.0%	(39.7–48.4%)	44.2%	(37.9–50.7%)	
Middle aged (45–64)	31.0%	(28.0–34.1%)	31.8%	(28.9–34.8%)	27.8%	(21.3–35.5%)	
Aged (+65)	12.5%	(9.8–15.7%)	12.9%	(10.1–16.4%)	10.8%	(7.5–15.3%)	
Wealth							0.00
Quintile 1 – Poorest	21.2%	(15.5–28.3%)	19.3%	(13.6–26.6%)	28.4%	(21.1–37.1%)	
Quintile 2	19.3%	(16.3–22.6%)	18.2%	(15.7–21.1%)	23.1%	(17.0–30.6%)	
Quintile 3	18.7%	(16.2–21.5%)	18.8%	(16.2–21.6%)	18.6%	(13.7–24.8%)	
Quintile 4	18.1%	(15.3–21.4%)	19.1%	(16.1–22.5%)	14.6%	(10.2–20.3%)	
Quintile 5 – Wealthiest	22.7%	(17.5–28.8%)	24.6%	(18.9–31.4%)	15.3%	(10.2–22.3%)	
Education							0.00
Literary course	3.1%	(2.2–4.4%)	3.1%	(2.0–4.7%)	3.3%	(1.6–6.7%)	
Primary	51.1%	(46.2–56.0%)	48.8%	(43.8–53.9%)	59.9%	(52.1–67.3%)	
Secondary	32.5%	(29.3–35.9%)	32.8%	(29.4–36.3%)	31.6%	(25.2–38.8%)	
University	13.2%	(10.6–16.3%)	15.3%	(12.3–19.0%)	5.1%	(3.1–8.4%)	
Ethnicity							0.08
Latino	49.0%	(36.99–61.19%)	50.5%	(37.9–63.1%)	43.4%	(31.1–56.6%)	
Creole	23.2%	(15.7–32.8%)	21.4%	(14.7–30.2%)	29.8%	(17.8–45.4%)	
Mayan	14.0%	(7.09–25.89%)	14.3%	(7.1–26.9%)	12.9%	(6.5–23.9%)	
Garifuna	7.1%	(2.91–16.51%)	7.0%	(2.8–16.5%)	7.5%	(3.0–17.5%)	
Mixed and others	6.6%	(3.83–11.19%)	6.7%	(3.6–11.9%)	6.4%	(3.5–11.4%)	
Health Conditions2							0.87
No	77.4%	(74.3–80.2%)	78.0%	(72.3–82.8%)	77.2%	(73.9–80.3%)	
Yes	22.6%	(19.8–25.7%)	22.0%	(17.3–27.7%)	22.8%	(19.7–26.1%)	
District							0.23
Belize	19.4%	(9.5–35.4%)	18.0%	(8.6–33.7%)	24.6%	(11.2–45.8%)	
Cayo	19.2%	(8.66–37.39%)	19.5%	(8.8–38.0%)	18.1%	(7.7–36.9%)	
Corozal	16.6%	(6.90–34.98%)	17.2%	(7.1–36.2%)	14.5%	(5.3–33.9%)	
Orange Walk	15.6%	(6.46–33.20%)	15.4%	(6.3–33.1%)	16.6%	(6.2–37.4%)	
Stann Creek	16.1%	(6.65–34.10%)	16.9%	(7.0–35.5%)	13.2%	(5.1–29.9%)	
Toledo	13.1%	(5.52–27.83%)	13.1%	(5.5–28.2%)	12.9%	(5.2–28.4%)	
N	1261		1000		261		

1. Chi–square test for the difference in the distribution between the vaccinated and unvaccinated groups. 2. Health conditions include any of the following HIB, Hepatitis B, Hepatitis C, Tuberculosis, Hypertension, Diabetes, Obesity or overweight, Chronic kidney disease, Cancer, Cardiovascular disease, Asthma, Chronic obstructive pulmonary disease.

### Vaccination intentions, attitudes, and beliefs

We found significant differences in most behavioral variables (see Table 2). The largest difference was in self-efficacy: while 96.4% [95% CI:95.1–97.8%] of the vaccinated individuals considered it easy or very easy to get the vaccine, only 43.4% [95% CI 35.0–51.8%] of the unvaccinated shared the same views.

**Table 2:** Behavioral factors among vaccinated and unvaccinated people in Belize, Unweighted (September–October 2021).

Behavioral factors	All		Vaccinated		Unvaccinated		p-value
	%	95% CI	%	95% CI	%	95% CI	(Chi–square1)
Have you been vaccinated against COVID–19?							
Yes	79.3%	(77.0–81.5%)					

*Intentions*							
If the COVID-19 vaccine is available to you, how likely are you to get vaccinated?							
Definitely get vaccinated					34.5%	(28.9–40.5%)	
Probably get vaccinated					15.3%	(11.4–20.2%)	
Probably would not get vaccinated					10.7%	(7.5–15.1%)	
Definitely would not get vaccinated					31.0%	(25.7–36.9%)	
Does not know					8.4%	(5.6–12.5%)	

*Behavioral beliefs*							
You will get COVID–19							0.12
Likely / Very Likely	60.9%	(57.4–64.3%)	62.2%	(58.6–65.7%)	56.0%	(48.9–62.8%)	
Unlikely / Very Unlikely	26.8%	(23.2–30.7%)	26.1%	(22.5–30.1%)	29.2%	(23.0–36.3%)	
Does not know	12.3%	(9.8–15.3%)	11.7%	(8.8–15.3%)	14.8%	(10.2–20.9%)	
A friend, family member or coworker will get COVID–19							0.31
Likely / Very Likely	73.9%	(70.9–76.8%)	74.5%	(71.0–77.7%)	71.8%	(66.3–76.8%)	
Unlikely / Very Unlikely	13.3%	(10.6–16.5%)	12.6%	(9.8–16.0%)	15.8%	(11.3–21.8%)	
Does not know	12.8%	(10.8–15.1%)	12.9%	(10.8–15.4%)	12.3%	(8.4–17.7%)	
You will have to go to a hospital if you get COVID–19							0.77
Likely / Very Likely	49.1%	(44.1–54.1%)	49.2%	(43.9–54.5%)	48.8%	(41.7–55.9%)	
Unlikely / Very Unlikely	40.6%	(36.3–45.2%)	40.3%	(35.8–44.9%)	42.0%	(35.2–49.0%)	
Does not know	10.3%	(8.1–12.9%)	10.5%	(8.1–13.5%)	9.3%	(6.8–12.4%)	
You will need to quarantine even if you don't get COVID–19							0.74
Likely/Very Likely	81.4%	(76.9–85.2%)	81.6%	(76.7–85.6%)	80.8%	(74.2–86.1%)	
Unlikely/Very Unlikely	15.4%	(11.9–19.6%)	15.4%	(11.8–19.8%)	15.4%	(10.9–21.4%)	
Does not know	3.2%	(1.9–5.2%)	3.1%	(1.7–5.3%)	3.7%	(1.7–7.9%)	
You will get COVID–19 and infect someone else							0.05
Likely/Very Likely	53.9%	(49.0–58.7%)	55.6%	(50.9–60.3%)	47.2%	(39.0–55.6%)	
Unlikely / Very Unlikely	39.1%	(33.9–44.6%)	37.8%	(32.7–43.3%)	43.8%	(34.7–53.3%)	
Does not know	7.1%	(4.7–10.4%)	6.5%	(4.1–10.2%)	9.0%	(5.5–14.3%)	
A friend, family member or coworker will die of COVID–19							0.00
Likely/Very Likely	61.4%	(57.5–65.1%)	63.6%	(59.6–67.4%)	53.0%	(46.0–59.9%)	
Unlikely/Very Unlikely	14.8%	(11.8–18.4%)	13.1%	(10.4–16.3%)	21.6%	(15.7–28.9%)	
Does not know	23.8%	(20.1–28.0%)	23.4%	(19.7–27.5%)	25.4%	(19.3–32.8%)	

*Attitudes*							
How safe do you think a COVID-19 vaccine is for you? Would you say… Moderately/Very Safe	66.0%	(62.2–69.9%)	76.1%	(72.7–79.4%)	27.6%	(20.5–34.7%)	0.00
How much protection do you think a COVID-19 vaccine would give you from getting ill-? Protect Moderately/A lot	60.8%	(56.3–65.4%)	70.2%	(65.8–74.5%)	25.3%	(19.2–31.4%)	0.00
How concerned are you that the COVID-19 vaccine might not be safe? Slightly/Somewhat concerned	72.6%	(68.5–76.7%)	80.7%	(76.5–85.0%)	41.4%	(33.8–49.0%)	0.00

*Normative beliefs*							
Do you think most of your friends and family will get a COVID-19 vaccine, if it is recommended for them?							0.00
Most/All of them	64.3%	(59.7–68.6%)	69.7%	(65.5–73.7%)	43.4%	(35.1–52.0%)	
Some/None of them	28.3%	(24.1–32.9%)	24.4%	(20.6–28.7%)	42.9%	(34.2–52.0%)	
Does not know	7.4%	(5.8–9.6%)	5.8%	(4.2–8.1%)	13.7%	(9.9–18.7%)	
Do you think most people in your community will get a COVID-19 vaccine, if it is recommended for them?							0.00
Most/All of them	41.3%	(36.5–46.3%)	46.1%	(41.2–51.0%)	23.3%	(17.6–30.2%)	
Some/None of them	40.5%	(36.4–44.7%)	38.5%	(34.3–42.9%)	48.4%	(40.8–56.0%)	
Does not know	18.1%	(14.6–22.3%)	15.5%	(11.9–19.8%)	28.3%	(23.1–34.2%)	

*Perceived norms*							
My parents and/or siblings think that I should get vaccinated against COVID–19							0.00
Somewhat/Strongly Agree	68.1%	(64.8–71.2%)	73.6%	(70.2–76.7%)	47.1%	(40.3–54.1%)	
Somewhat/Strongly Disagree	26.0%	(22.9–29.5%)	20.1%	(17.1–23.6%)	48.6%	(41.7–55.6%)	
Does not know	5.9%	(4.4–7.8%)	6.3%	(4.6–8.4%)	4.3%	(2.4–7.5%)	
My close friends think that I should get vaccinated against COVID–19							0.00
Somewhat/Strongly Agree	65.4%	(62.1–68.6%)	70.5%	(66.8–73.9%)	46.0%	(38.7–53.4%)	
Somewhat/Strongly Disagree	30.1%	(27.1–33.2%)	24.8%	(21.7–28.3%)	50.2%	(42.8–57.6%)	
Does not know	4.5%	(3.2–6.3%)	4.7%	(3.2–6.8%)	3.8%	(2.0–7.1%)	
People in my community believe that everyone should be vaccinated against COVID–19							0.00
Somewhat/Strongly Agree	47.6%	(43.3–51.9%)	51.9%	(48.1–55.6%)	31.2%	(24.0–39.4%)	
Somewhat/Strongly Disagree	40.7%	(36.8–44.6%)	36.1%	(32.4–39.9%)	58.1%	(50.1–65.7%)	
Does not know	11.8%	(9.3–14.8%)	12.0%	(9.3–15.5%)	10.7%	(7.3–15.4%)	
Doctors and nurses think that everyone who has no medical problems should get the vaccine							0.00
Somewhat/Strongly Agree	62.2%	(57.9–66.3%)	67.2%	(62.7–71.3%)	43.5%	(35.5–51.8%)	
Somewhat/Strongly Disagree	28.9%	(25.0–33.1%)	24.3%	(20.8–28.2%)	46.2%	(37.6–55.0%)	
Does not know	8.9%	(6.9–11.4%)	8.5%	(6.3–11.5%)	10.3%	(7.1–14.7%)	

*Efficacy beliefs*							
What would motivate you to get vaccinated?							
Protect my health	61.9%	(57.8–66.0%)	68.9%	(64.5–73.3%)	35.3%	(28.3–42.2%)	0.00
Protect health of others	53.3%	(48.0–58.6%)	57.6%	(51.6–63.5%)	36.9%	(29.6–44.2%)	0.00
Look for employment, work or go back to school	33.0%	(29.2–36.8%)	36.2%	(31.7–40.7%)	20.7%	(15.1–26.4%)	0.00
Social activities, traveling, government mandate or encouraged by others	15.3%	(12.5–18.1%)	16.7%	(13.2–20.3%)	9.9%	(7.0–12.9%)	0.01
Nothing, freedom or no choice	2.1%	(1.3–2.9%)	0.7%	(0.2–1.1%)	7.6%	(4.1–11.1%)	0.00

*Self-efficacy*							
How easy do you think it will be / was to get a COVID-19 vaccine for yourself? Would you say…Somewhat/Very easy	85.4%	(83.0–87.8%)	96.4%	(95.1–97.8%)	43.4%	(35.0–51.8%)	0.00
N	1261		1000		261		

1. Chi–square test for the difference in the distribution between the vaccinated and unvaccinated groups.

Moreover, we identified significant differences in attitudes towards vaccination. Among the vaccinated, 76.1% [95% Confidence Interval (CI): 72.7–79.4%] believed the vaccine was safe and 70.2% [95% CI: 65.8–74.5%] believed that it protected them from sickness. In contrast, these beliefs were held only by 27.6% [95% CI: 20.5–34.7%] and 25.3% [95% CI: 19.2–31.4%%] of the unvaccinated respectively. As for behavioral beliefs, 47.2% [95% CI: 39.0–55.6%] of the unvaccinated believed they would get COVID-19 and infect someone else, compared with 55.6% [95% CI: 50.9–60.3%] of the vaccinated. However, we did not find significant differences between groups considering the risk of getting COVID-19; a friend, family member or coworker getting COVID-19, or the need for quarantine. Interestingly, almost half of the individuals in both groups believe they may require hospitalization if they contract COVID-19.

Regarding efficacy beliefs, although a smaller proportion of the unvaccinated identified reasons that motivated them to get vaccinated, motivations were similarly distributed between both groups. The main motivations were to protect their health and the health of others. In terms of perceived norms, while approximately two thirds of the vaccinated believe their parents, siblings, close friends, nurses and doctors think they should get vaccinated, whereas only about 40% of the unvaccinated believed the same. Nevertheless, among the vaccinated, only half believed that people in their community would support vaccination. Similar results were observed for normative beliefs, where a majority of the vaccinated believed most of their friends and family would get vaccinated, while less than half of the unvaccinated shared this belief.

### Reasons for not getting vaccinated

Among individuals who said they would probably not or definitely not get vaccinated (41.7%), the primary reason for not getting vaccinated was fear of side effects, accounting for 48.6% among this group (see Table 3). Of those who reported fear of side effects, more than half reported preexisting health conditions (see Table 4), and some explicitly mentioned these conditions as the reason for not getting vaccinated. Almost one third of the unvaccinated did not trust vaccines, a sentiment more commonly expressed among younger respondents (see Table 5).

**Table 3:** Reasons for not getting the COVID–19 vaccine among the unvaccinated, Unweighted (September–October 2021).

Why would you not get a COVID–19 vaccine?	%	95% CI
I am concerned about side effects of the COVID–19 vaccine	48.6%	(38.9**–**58.4%)
I don't trust COVID–19 vaccines.	32.1%	(23.5**–**41.7%)
Does not know if a COVID–19 vaccine will work.	17.4%	(10.8**–**25.9%)
I don't think I need a COVID–19 vaccine.	16.5%	(10.1**–**24.8%)
I want to wait to see if it is safe and maybe get vaccinated later.	9.2%	(4.5**–**16.2%)
Other reason	9.2%	(4.5**–**16.2%)
I don't like vaccines	7.3%	(3.2**–**14.0%)
I do not trust the government	5.5%	(2.0**–**11.6%)
Because of my religious beliefs.	3.7%	(1.0**–**9.1%)
I don't trust the COVID–19 vaccine being offered.	2.8%	(0.6**–**7.8%)
I have heard negative information about vaccines in the media.	2.8%	(0.6**–**7.8%)
My doctor has not recommended that I get the COVID–19 vaccine.	1.8%	(0.2**–**6.5%)
Does not know	1.8%	(0.2**–**6.5%)
**N**	109	

Note: Question asked only to unvaccinated people who said they would *probably not* or *definitely not* get vaccinated if the vaccine was available for them. Multiple responses were allowed. For option Other, people could specify an open-ended response. Open-ended responses were manually codified into the different categories when possible. Otherwise, they were coded as “Other reason”.

**Table 4:** Reasons for not getting the COVID–19 vaccine among the unvaccinated, by health condition, Unweighted (September–October 2021).

Why would you not get a COVID–19 vaccine?	No health conditions		Health conditions1	
	%	95% CI	%	95% CI
I am concerned about side effects of the COVID–19 vaccine	46.2%	(34.8–57.8%)	54.8%	(36.0–72.7%)
I don't trust COVID–19 vaccines.	34.6%	(24.2–46.2%)	25.8%	(11.9–44.6%)
I don't think I need a COVID–19 vaccine.	14.1%	(7.3–23.8%)	22.6%	(9.6–41.1%)
Does not know if a COVID–19 vaccine will work.	16.7%	(9.2–26.8%)	19.4%	(7.5–37.5%)
I do not trust the government	2.6%	(0.3–9.0%)	12.9%	(3.6–29.8%)
I don't like vaccines	6.4%	(2.1–14.3%)	9.7%	(2.0–25.8%)
I want to wait to see if it is safe and maybe get vaccinated later.	9.0%	(3.7–17.6%)	9.7%	(2.0–25.8%)
Other reason	10.3%	(4.5–19.2%)	6.5%	(0.8–21.4%)
I don't trust the COVID–19 vaccine being offered.	2.6%	(0.3–9.0%)	3.2%	(0.1–16.7%)
Because of my religious beliefs.	3.8%	(0.8–10.8%)	3.2%	(0.1–16.7%)
I have heard negative information about vaccines in the media.	2.6%	(0.3–9.0%)	3.2%	(0.1–16.7%)
My doctor has not recommended that I get the COVID–19 vaccine.	2.6%	(0.3–9.0%)	0.0%	(0.0–11.2%)
Does not know	2.6%	(0.3–9.0%)	0.0%	(0.0–11.2%)
N	78		31	

Note: Question asked only to unvaccinated people who said they would *probably not* or *definitely not* get vaccinated if the vaccine was available for them. Multiple responses were allowed. For option other, people could specify a response. These responses were codified into the different categories when available. Otherwise, they were coded as “Other reason”. 1. Health conditions include any of the following HIB, Hepatitis B, Hepatitis C, Tuberculosis, Hypertension, Diabetes, Obesity or overweight, Chronic kidney disease, Cancer, Cardiovascular disease, Asthma, Chronic obstructive pulmonary disease.

**Table 5:** Reasons for not getting the COVID–19 vaccine among the unvaccinated, by MeSH age groups, Unweighted (September–October 2021).

Why would you not get a COVID–19 vaccine?	Adolescents and young adults (15–24)		Adults (25–44)		Middle aged (45–64)		Aged (+65)	
	%	95% CI	%	95% CI	%	95% CI	%	95% CI
I am concerned about side effects of the COVID–19 vaccine	31.3%	(11.0–58.7%)	60.5%	(44.4–75.0%)	50.0%	(32.9–67.1%)	28.6%	(8.4–58.1%)
Does not know if a COVID–19 vaccine will work.	18.8%	(4.0–45.6%)	18.6%	(8.4–33.4%)	19.4%	(8.2–36.0%)	7.1%	(0.2–33.9%)
I don't think I need a COVID–19 vaccine.	12.5%	(1.6–38.3%)	20.9%	(10.0–36.0%)	11.1%	(3.1–26.1%)	21.4%	(4.7–50.8%)
I don't like vaccines	6.3%	(0.2–30.2%)	7.0%	(1.5–19.1%)	8.3%	(1.8–22.5%)	7.1%	(0.2–33.9%)
My doctor has not recommended that I get the COVID–19 vaccine.	0.0%	(0.0–20.6%)	0.0%	(0.0–8.2%)	5.6%	(0.7–18.7%)	0.0%	(0.0–23.2%)
I want to wait to see if it is safe and maybe get vaccinated later.	12.5%	(1.6–38.3%)	9.3%	(2.6–22.1%)	8.3%	(1.8–22.5%)	7.1%	(0.2–33.9%)
I don't trust COVID–19 vaccines.	43.8%	(19.8–70.1%)	44.2%	(29.1–60.1%)	16.7%	(6.4–32.8%)	21.4%	(4.7–50.8%)
I don't trust the COVID–19 vaccine being offered.	0.0%	(0.0–20.6%)	2.3%	(0.1–12.3%)	5.6%	(0.7–18.7%)	0.0%	(0.0–23.2%)
I do not trust the government	0.0%	(0.0–20.6%)	9.3%	(2.6–22.1%)	5.6%	(0.7–18.7%)	0.0%	(0.0–23.2%)
Because of my religious beliefs.	6.3%	(0.2–30.2%)	0.0%	(0.0–8.2%)	8.3%	(1.8–22.5%)	0.0%	(0.0–23.2%)
I have heard negative information about vaccines in the media.	0.0%	(0.0–20.6%)	4.7%	(0.6–15.8%)	2.8%	(0.1–14.5%)	0.0%	(0.0–23.2%)
Other reason	6.3%	(0.2–30.2%)	11.6%	(3.9–25.1%)	2.8%	(0.1–14.5%)	21.4%	(4.7–50.8%)
Does not know	0.0%	(0.0–20.6%)	2.3%	(0.1–12.3%)	2.8%	(0.1–14.5%)	0.0%	(0.0–23.2%)
**N**	16		43		36		14	

Note: Question asked only to unvaccinated people who said they would *probably not* or *definitely not* get vaccinated if the vaccine was available for them. Multiple responses were allowed. For option other, people could specify a response. These responses were codified into the different categories when available. Otherwise, they were coded as “Other reason”.

### Factors associated with COVID-19 vaccine uptake

People over 65 years were nearly two and a half times more likely to be vaccinated (Odds Ratio [OR] 2.56 [95% CI: 1.14–5.74]) compared to other age groups (see Table 6: Predictors of vaccine uptake). Additionally, individuals living in Stann Creek were 3.16 times [OR 95% CI: 1.41–7.08] more likely to be vaccinated compared to those living in other districts. When accounting for other variables, behavioral beliefs were not statistically significant.

**Table 6:** Logistic regression analyses of factors associated with vaccine uptake among all respondents, and with intention to get vaccinated among the unvaccinated in Belize, Unweighted (September–October 2021).

	Predictors of vaccine uptake		Predictors of intention to get vaccinated	
	Odds ratio	95% CI	Odds ratio	95% CI
Female	0.84	[0.55,1.29]	2.26	[0.76,6.73]

Age groups				
Adolescents and Young Adults (15–24)	Ref.		Ref.	
Adults (25–44)	1.66	[0.95,2.89]	0.36	[0.09,1.38]
Middle aged (45–64)	1.74	[0.93,3.25]	0.35	[0.07,1.80]
Aged (65 + )	2.56*	[1.14,5.74]	0.75	[0.09,6.11]
Poorest 40%	0.68	[0.44,1.04]	1.01	[0.33,3.08]

Ethnicity				
Creole	Ref.		Ref.	
Garifuna	0.43	[0.16,1.15]	15.55*	[1.64,147.26]
Mixed, Others, DK	0.49	[0.21,1.17]	0.86	[0.06,12.02]
Mayan	2.12	[0.86,5.21]	1.71	[0.15,19.37]
Latino	1.45	[0.83,2.51]	2.26	[0.60,8.46]
Health conditions	1.2	[0.72,2.02]	0.43	[0.10,1.83]

District				
Belize	Ref.		Ref.	
Cayo	1.4	[0.73,2.68]	0.26	[0.05,1.46]
Corozal	1.34	[0.66,2.75]	0.29	[0.04,2.00]
Orange Walk	0.88	[0.43,1.82]	0.34	[0.06,2.07]
Stann Creek	3.16**	[1.41,7.08]	0.33	[0.04,2.39]
Toledo	1.95	[0.75,5.04]	0.62	[0.03,11.02]

You will get COVID–19 and infect someone else				
Very Unlikely/Unlikely	Ref.		Ref.	
Likely/Very Likely/Already happened	0.94	[0.60,1.48]	0.76	[0.21,2.73]
Does not know	0.64	[0.28,1.46]	0.25	[0.03,2.16]

A friend, family member or coworker will get COVID–19 and die				
Very Unlikely/Unlikely	Ref.		Ref.	
Likely/Very Likely/Already happened	1.09	[0.62,1.91]	0.59	[0.14,2.42]
Does not know	1.37	[0.71,2.65]	2.31	[0.42,12.69]

Do you think most of your friends and family will get a COVID–19 vaccine?				
Some/None of them	ref.		ref.	
Most/All of them	1.39	[0.86,2.26]	1.82	[0.50,6.65]
Does not know	3.50**	[1.43,8.57]	0.63	[0.09,4.72]

Do you think most of the people in your community will get a COVID–19 vaccine?				
Some/None of them	ref.		ref.	
Most/All of them	1.46	[0.87,2.43]	1.16	[0.30,4.57]
Does not know	0.56	[0.31,1.04]	0.95	[0.22,4.05]

What would/has motivated you to get the COVID–19 vaccine?				
Protect my health	2.46***	[1.61,3.76]	45.17***	[10.89,187.39]
Look for employment, work or go back to school	2.02**	[1.28,3.20]	12.52***	[3.28,47.82]
Social activities, traveling, government mandate or encouraged by others	1.54	[0.85,2.81]	27.86***	[4.67,166.17]
Nothing, freedom or no option	0.57	[0.14,2.28]	0.52	[0.03,7.88]

How safe do you think a COVID-19 vaccine is for you?				
Moderately/Very Safe	2.57***	[1.52,4.35]	4.26	[0.81,22.50]

How much protection do you think a COVID-19 vaccine would give you from getting ill-?				
Protect Moderately / A lot	1.78*	[1.04,3.03]	6.79*	[1.03,44.71]

My parents and/or siblings think that I should get vaccinated against COVID–19				
Strongly/Somewhat disagree/Neither	Ref.		Ref.	
Somewhat/Strongly agree	1.62	[0.97,2.69]	1.29	[0.39,4.26]
Does not know	3.01*	[1.08,8.42]	1.53	[0.09,26.82]

People in my community think that everyone should get vaccinated against COVID–19				
Strongly/Somewhat disagree/Neither	Ref.		Ref.	
Somewhat/Strongly agree	0.83	[0.50,1.37]	2.01	[0.58,6.94]
Does not know	0.56	[0.27,1.15]	1.81	[0.37,8.98]

How easy do you think it will be / was to get a COVID-19 vaccine for yourself? Would you say…				
Somewhat / Very easy	23.63***	[14.21,39.27]	2.55	[0.80,8.07]
Constant	0.02***	[0.01,0.05]	0.07*	[0.01,0.63]
N	1261		261	

* p < 0.05, ** p < 0.01, *** p < 0.001.

We found that efficacy beliefs, self-efficacy and vaccine attitudes were associated with increased likelihood of vaccination. People who believed it was easy to get a vaccine were over 23 times more likely to be vaccinated (OR 23.63 [95% CI: 14.21–39.27]), underlining accessibility as an important barrier. Moreover, those who believed in the vaccine safety were 2.57 times [OR 95% CI: 1.52–4.35] more likely to be vaccinated, and those who thought the vaccine would protect them were 1.78 times more likely to be vaccinated [OR 95% CI: 1.04–3.03]. Accordingly, respondents who expressed willingness to get vaccinated to protect their health, return to school, go back to work, or find employment were more likely to be vaccinated. Perceived norms also played a role, as individuals who were uncertain if their parents and/or siblings though they should get vaccinated were 3.01 times [OR 95% CI: 1.08–8.42] more likely to be vaccinated. Concerning normative beliefs, we found that while uncertainty about parents or friends getting vaccinated increased the likelihood of getting vaccinated, uncertainty about people in the community getting vaccinated decreased the likelihood.

When analyzing data for male and female responses separately, the results for most behavioral variables are consistent (see Table 7). For both genders, efficacy beliefs, self-efficacy and vaccine attitudes were associated with increased likelihood of vaccination. Nevertheless, while vaccine safety increased the likelihood of vaccination for female and male respondents, male respondents were also more likely to be vaccinated if they thought the vaccine was effective, which was not the case for female respondents. Moreover, among female respondents, adolescents and young adults (15–24 years) were less likely to be vaccinated compared to other age groups. In contrast, male respondents did not show significant differences by age. Additionally, male respondents from the two lowest quintiles were less likely to be vaccinated.

**Table 7:** Logistic regression analyses of factors associated with vaccine uptake among all respondents, by gender in Belize, Unweighted (September–October 2021).

Predictors of vaccine uptake	Female (67%)		Male (33%)	
	Odds ratio	95% CI	Odds ratio	95% CI
Age groups				
Adolescents and Young Adults (15–24)	Ref.		Ref.	
Adults (25–44)	1.95*	[1.00,3.79]	1.62	[0.50,5.27]
Middle aged (45–64)	2.79*	[1.26,6.19]	0.57	[0.16,1.99]
Aged (65 + )	4.38**	[1.51,12.71]	0.55	[0.11,2.66]
Poorest 40%	0.84	[0.50,1.41]	0.34*	[0.13,0.89]

Ethnicity				
Creole	Ref.		Ref.	
Garifuna	0.41	[0.12,1.35]	0.42	[0.05,3.31]
Mixed, Others, DK	0.62	[0.21,1.86]	0.29	[0.05,1.58]
Mayan	1.76	[0.61,5.09]	4.28	[0.54,33.76]
Latino	1.13	[0.57,2.23]	3.32*	[1.06,10.39]
Health conditions	1.35	[0.72,2.56]	0.72	[0.25,2.09]

District				
Belize	Ref.		Ref.	
Cayo	1.53	[0.70,3.37]	0.83	[0.20,3.38]
Corozal	1.6	[0.66,3.86]	0.69	[0.16,2.93]
Orange Walk	1.37	[0.57,3.29]	0.16*	[0.03,0.87]
Stann Creek	3.65*	[1.34,9.92]	2.08	[0.41,10.46]
Toledo	1.84	[0.61,5.59]	3.27	[0.33,32.33]

You will get COVID–19 and infect someone else				
Very Unlikely/Unlikely	ref.		ref.	
Likely/Very Likely/Already happened	1.2	[0.69,2.08]	0.4	[0.15,1.06]
Does not know	0.82	[0.30,2.24]	0.15*	[0.03,0.88]

A friend, family member or coworker will get COVID–19 and die				
Very Unlikely/Unlikely	ref.		ref.	
Likely/Very Likely/Already happened	1.08	[0.55,2.14]	1.13	[0.36,3.53]
Does not know	1.41	[0.64,3.09]	1.82	[0.46,7.14]

Do you think most of your friends and family will get a COVID–19 vaccine?				
Some/None of them	ref.		ref.	
Most/All of them	1.51	[0.85,2.69]	1.08	[0.38,3.06]
Does not know	2.48	[0.85,7.25]	20.82**	[2.88,150.60]

Do you think most of the people in your community will get a COVID–19 vaccine?				
Some/None of them	ref.		ref.	
Most/All of them	1.34	[0.72,2.47]	1.65	[0.57,4.76]
Does not know	0.67	[0.32,1.39]	0.24*	[0.06,0.95]

What would/has motivated you to get the COVID–19 vaccine?				
Protect my health	2.16**	[1.29,3.62]	4.01**	[1.57,10.23]
Look for employment, work or go back to school	2.18**	[1.23,3.88]	2.14	[0.87,5.26]
Social activities, traveling, government mandate or encouraged by others	1.57	[0.77,3.21]	1.09	[0.30,3.94]
Nothing, freedom or no option	1.06	[0.17,6.54]	0.32	[0.03,3.26]

How safe do you think a COVID-19 vaccine is for you?				
Moderately/Very Safe	2.34**	[1.24,4.43]	5.06**	[1.50,17.04]

How much protection do you think a COVID-19 vaccine would give you from getting ill-?				
Protect Moderately/A lot	1.42	[0.75,2.68]	4.19*	[1.25,14.11]

My parents and/or siblings think that I should get vaccinated against COVID–19				
Strongly/Somewhat disagree/Neither	ref.		ref.	
Somewhat/Strongly agree	1.81	[0.96,3.40]	1.74	[0.59,5.10]
Does not know	2.38	[0.66,8.57]	6.36	[0.71,56.93]

People in my community think that everyone should get vaccinated against COVID–19				
Strongly/Somewhat disagree/Neither	ref.		ref.	
Somewhat/Strongly agree	0.73	[0.39,1.36]	1.23	[0.44,3.41]
Does not know	0.54	[0.22,1.34]	0.85	[0.21,3.55]

How easy do you think it will be / was to get a COVID-19 vaccine for yourself? Would you say…				
Somewhat / Very easy	23.63***	[14.34,53.05]	22.15***	[7.63,64.31]
Constant	0.01***	[0.00,0.04]	0.03***	[0.00,0.24]
N	845		416	

* p < 0.05, ** p < 0.01, *** p < 0.001.

### Factors associated with vaccination intention among the unvaccinated

Among the unvaccinated, we observed that individuals who self-identified as Garifuna were more likely to intend getting vaccinated compared to those of other ethnicities (see Table 2: Predictors of intention to get vaccinated). Efficacy beliefs played a significant role, as people who were motivated to protect their health, continue work or education, or resume social activities were many times more likely to intend getting vaccinated. Further, positive attitudes towards the vaccine, particularly the belief that the vaccine would offer them protection, also increased the likelihood of vaccination. Those who believed that vaccines offered them protection were over 6 times (OR 6.79 [95% CI: 1.03–44.71]) more likely to intend getting vaccinated.

## Discussion

To our knowledge, this is the first study describing factors associated with COVID-19 vaccine uptake and vaccine hesitancy in Belize. Our findings contribute to the description of COVID-19 vaccination uptake worldwide –data for Belize was not previously available [29]. Our results indicate that Belize’s MOHW has been successful vaccinating a large proportion of the eligible population. However, accessibility has been identified as a major limitation to further increasing vaccine coverage. Targeting adolescents and young adults and addressing concerns about side effects pose additional challenges. We also found that approximately one third of the unvaccinated individuals are strong vaccine refusers and deniers, constituting 6.6% of the total population, making it very difficult to change their minds regarding vaccination.

Our study shows that about one third of the unvaccinated individuals expressed willingness to get vaccinated. Enhancing self-efficacy by advertising vaccination events and making vaccines more accessible to communities could tip the balance towards vaccination. Despite individuals with preexisting conditions being at higher risk of COVID-19 complications, fear of side effects prevented many of them from getting vaccinated. The MOHW should specifically address side-effects in their communication strategy, including face-to-face interactions with health personnel. Furthermore, individuals with preexisting conditions should be specifically targeted. As demonstrated in vaccination efforts for other diseases [30], every interaction with healthcare workers should be considered an opportunity to promote COVID-19 vaccination.

Behavioral beliefs were not found to be associated with increased vaccine uptake, as the perceived risk of COVID-19 was similarly shared between vaccinated and unvaccinated groups. However, attitudes towards vaccination were drastically different between groups. In addition to side effects, some responses reflect common themes from vaccine deniers, including ideas related to natural prevention, conspiracy theories, and misrepresentation [31]. Studies have shown that even a small proportion of vaccine deniers can influence hesitant individuals [31]. Therefore, further study is needed to understand whether these ideas have penetrated the general population and to determine the most effective approaches to debunk them.

Many of our findings align with previous studies. Other research has also identified protecting personal health and the health of others as the primary reason for vaccination [8]. The importance of community support and trust in government and health authorities have also been associated with vaccine uptake [9], [10]. Communicating vaccine coverage, particularly emphasizing that the majority of people in Belize have chosen vaccination, could reinforce normative beliefs and perceived norms, and encourage undecided individuals.

Regarding age-specific differences, young adults and adolescents have been found to exhibit more vaccine hesitancy than older age groups [7], [10]. The rollout of vaccines for adolescents in Belize occurred approximately seven months after the vaccines were made available for individuals over 60 years old (the first eligible group), which may partly explain differences in coverage, especially for female respondents. However, lack of trust in vaccines is also higher among the unvaccinated in these age groups.

Belize has a large proportion of young population, with approximately 33% (under 12 years old) not yet eligible for vaccination at the time of the surveys. According to World Health Organization recommendations, healthy children and adolescents were the lowest priority group for vaccination as of April 2022. Nevertheless, given the age-structure in Belize and the progress already achieved, vaccinating children could improve herd immunity and prevent COVID-19 related complications and deaths among children [32]. In May 2022, Belize began rolling-out vaccines for 5–12-year-olds. The challenge for Belize in the future is ensuring that COVID vaccines are continuously available for all eligible age-groups and not relying on sporadic donations, as has been the case thus far [33], [34].

While our study offers valuable insights, it has some limitations. Our responses are based on self-reported vaccination coverage, which are subject to social desirability and recall biases. However, as COVID-19 vaccination is a salient and recent event, we believe recall bias is minimized. Two small Mennonite communities in the study rejected data collection and had to be replaced, potentially causing marginal bias, together representing about 2% of the population. Overall, we do not believe there are large pockets of unvaccinated communities in the country. For our analysis, we only considered a general question on vaccination, regardless of the type of vaccine or doses received. Future studies could investigate factors associated with complete vaccination. Lastly, due to the pandemic, the survey training, pilot, and quality controls for the survey were conducted remotely. Although there are no indications of major quality problems, we acknowledge that face-to-face interactions might offer additional benefits.

Since the preliminary results became available in November 2021, the MOHW has taken important steps to strengthen the vaccination strategy. First, it conducted a follow-up qualitative study to learn about the specific reasons for vaccine hesitance. Second, the MOHW improved the messages and visuals of the communications campaign and launched a digital campaign targeting young people. For example, emphasizing vaccine safety for people with preexisting health conditions, presenting age-adjusted mortality rates instead of crude death counts, and representing all ethnic groups in communication materials. Third, it increased the hours of operation of vaccination posts, provided options for weekends, and improved dissemination of vaccination events. As part of these efforts, the MOHW has also tapped the high coverage of mobile networks and disseminated information through SMS.

## Conclusions

Belize has achieved considerable progress vaccinating eligible people against COVID-19, over four fifths of the eligible population were vaccinated at the time of this study. The data shows that accessibility has been the primary factor limiting the increase in vaccine coverage. Our study indicates that <7% of the eligible population in Belize have been strong vaccine deniers, which is an encouraging finding. However, the challenge of reaching the last mile remains. While increasing vaccine awareness could have been sufficient at first, reaching the few pending vaccination would likely require additional efforts. To increase vaccine uptake increased outreach effort is needed to address access barriers. Nuanced messaging and close interpersonal communication will be necessary to persuade vaccine hesitant individuals. Our results call for increased efforts improving self-efficacy, efficacy beliefs, and perceived norms.

## Funding

This work was supported by the Bill & Melinda Gates Foundation [Grant Number OPPGH5328], the Carlos Slim Foundation, and the Government of Canada through the Salud Mesoamerica Initiative, as well as Technical Cooperation (BL-T1141) and Economic and Sector Work (RG-1814) resources from the Inter-American Development Bank. Funders had no role in study design, data collection and analysis, decision to publish, or preparation of the manuscript.

## Ethical approval and informed consent

Ethical approval for this study was received from the Institutional Review Board of Belize’s Ministry of Health and Wellness (Ref. IOR/39/01 vol II (32)). Verbal informed consent was obtained from each study participant. Participation was elective and participants could withdraw at any time.

## Declaration of Competing Interest

The authors declare that they have no known competing financial interests or personal relationships that could have appeared to influence the work reported in this paper. The opinions expressed in this publication are those of the authors and do not necessarily reflect the views of the Inter-American Development Bank, its Board of Directors, or the countries they represent. The authors report no conflicts of interest.

## Data Availability

Data will be made available on request.

## References

[1] Reuters. COVID-19 Global tracker. Ctries Report Most Doses Adm Popul; 2022. <https://graphics.reuters.com/world-coronavirus-tracker-and-maps/> [accessed February 9, 2022].

[2] Roser M., Ortiz-Ospina E. (2022). COVID-19 vaccine doses administered per 100 people. Our World Data.

[3] Anderson R.M., Vegvari C., Truscott J., Collyer B.S. (2020). Challenges in creating herd immunity to SARS-CoV-2 infection by mass vaccination. Lancet.

[4] Koritala K., Hussain A., Pleshkova Y., Dondapati L., Tirupathi R., Rabaan A.A. (2021). A narrative review of emergency use authorization versus full FDA approval and its effect on COVID-19 vaccination hesitancy. Infez Med.

[5] Dai H., Saccardo S., Han M.A., Roh L., Raja N., Vangala S. (2021). Behavioural nudges increase COVID-19 vaccinations. Nature.

[6] Bavel J.J.V., Baicker K., Boggio P.S., Capraro V., Cichocka A., Cikara M. (2020). Using social and behavioural science to support COVID-19 pandemic response. Nat Hum Behav.

[7] Kessels R., Luyten J., Tubeuf S. (2021). Willingness to get vaccinated against Covid-19 and attitudes toward vaccination in general. Vaccine.

[8] Kaplan R.M., Milstein A. (2021). Influence of a COVID-19 vaccine’s effectiveness and safety profile on vaccination acceptance. Proc Natl Acad Sci.

[9] Wakefield J.R.H., Khauser A. (2021). Doing it for us: Community identification predicts willingness to receive a COVID -19 vaccination via perceived sense of duty to the community. J Commun Appl Soc Psychol.

[10] Ahamed F., Ganesan S., James A., Zaher W.A. (2021). Understanding perception and acceptance of Sinopharm vaccine and vaccination against COVID–19 in the UAE. BMC Public Health.

[11] Chang T, Jacobson M, Shah M, Pramanik R, Shah S. Financial incentives and other nudges do not increase COVID-19 vaccinations among the vaccine hesitant. Cambridge, MA: National Bureau of Economic Research; 2021. <https://doi.org/10.3386/w29403>.

[12] Cerda A.A., García L.Y. (2021). Hesitation and refusal factors in individuals’ decision-making processes regarding a coronavirus disease 2019 vaccination. Front Public Health.

[13] Ceulemans M., Foulon V., Panchaud A., Winterfeld U., Pomar L., Lambelet V. (2021). Vaccine willingness and impact of the COVID-19 pandemic on women’s perinatal experiences and practices—a multinational, cross-sectional study covering the first wave of the pandemic. Int J Environ Res Public Health.

[14] Gencer H., Özkan S., Vardar O., Serçekuş P. (2021). The effects of the COVID 19 pandemic on vaccine decisions in pregnant women. Women Birth.

[15] Milkman K.L., Beshears J., Choi J.J., Laibson D., Madrian B.C. (2011). Using implementation intentions prompts to enhance influenza vaccination rates. Proc Natl Acad Sci.

[16] Sprengholz P., Henkel L., Betsch C. (2022). Payments and freedoms: effects of monetary and legal incentives on COVID-19 vaccination intentions in Germany. PLoS One.

[17] Ashworth M., Thunström L., Cherry T.L., Newbold S.C., Finnoff D.C. (2021). Emphasize personal health benefits to boost COVID-19 vaccination rates. Proc Natl Acad Sci.

[18] Santos H.C., Goren A., Chabris C.F., Meyer M.N. (2021). Effect of targeted behavioral science messages on COVID-19 vaccination registration among employees of a large health system: a randomized trial. JAMA Netw Open.

[19] Eichengreen B., Aksoy C.G., Saka O. (2021). Revenge of the experts: Will COVID-19 renew or diminish public trust in science?. J Public Econ.

[20] Fishbein M., Yzer M.C. (2003). Using theory to design effective health behavior interventions. Commun Theory.

[21] Rios-Zertuche D., Cuchilla J., Zúñiga-Brenes P., Hernández B., Jara P., Mokdad A.H. (2016). Alcohol abuse and other factors associated with risky sexual behaviors among adolescent students from the poorest areas in Costa Rica. Int J Public Health.

[22] Mokdad A.H., Colson K.E., Zúñiga-Brenes P., Ríos-Zertuche D., Palmisano E.B., Alfaro-Porras E. (2015). Initiative: design, implementation, and baseline findings. Popul Health Metr.

[23] WHO. Covid-19 Question Bank. World Health Organ Data Collect Tools 2021. <https://www.who.int/data/data-collection-tools/covid19-question-bank> [accessed June 25, 2021].

[24] CDC. Vaccine Confidence Survey Question Bank; 2021.https://www.cdc.gov/vaccines/covid-19/vaccinate-with-confidence/rca-guide/downloads/CDC_RCA_Guide_2021_Tools_AppendixD_Surveys-508.pdf.

[25] Metrics for Management (2018). The Equity Tool: Belize. EquityTool.

[26] Ruiz M.T., Verbrugge L.M. (1997). A two way view of gender bias in medicine. J Epidemiol Commun Health.

[27] Archer K.J., Lemeshow S. (2006). Goodness-of-fit test for a logistic regression model fitted using survey sample data. Stata J.

[28] Pregibon D. (1979).

[29] Babalola S, Krenn S, Rosen S, Serlemitsos E, Shaivitz M, Storey D, et al. COVID Behaviors Dashboard. Johns Hopkins Center for Communication Programs in collaboration with Facebook Data for Good, Delphi Group at Carnegie Mellon University, University of Maryland Social Data Science Center, Global Outbreak Alert and Response Network. Published September 2021. https://covidbehaviors.org/.

[30] Who (2019).

[31] Europe W.H.O. (2017).

[32] Malcangi G., Inchingolo A.D., Inchingolo A.M., Piras F., Settanni V., Garofoli G. (2022). COVID-19 infection in children and infants: current status on therapies and vaccines. Children.

[33] Ministry of Foreign Affairs, Mexico. Mexico donates 400,000 doses of COVID-19 vaccines to Belize, Bolivia and Paraguay. Press Release 269; 2021. <https://www.gob.mx/sre/prensa/mexico-donates-400-000-doses-of-covid-19-vaccines-to-belize-bolivia-and-paraguay?idiom=en> [accessed August 29, 2022].

[34] US Embassy in Belize (2021). The United States Donates an Additional 117,000 Pfizer Vaccines to Belize. News Events.

