# Supplementary material for: Factors associated with COVID-19 vaccination in Belize
Source: Vaccine X. 2023 Sep 1;15:100380. doi: 10.1016/j.jvacx.2023.100380 (PMC10483062; doi:10.1016/j.jvacx.2023.100380)
Supplement: Supplementary data 1 [file mmc1.pdf]

APPENDIX

Factors associated with COVID-19 vaccination in Belize

Rios-Zertuche, Diego; Giuliana Daga; Filippo Iorillo; Ana Mylena Aguilar Rivera;
Melissa Diaz -Musa; Natalia Largaespada Beer; Florencia López Boo; Julio Sabido.
Vaccine X.
https://doi.org/10.1016/j.jvacx.2023.100380

Table A1: Models with all covariates – Logistic regression analyses of factors associated with vaccine uptake among all respondents, and with intention to get vaccinated among the unvaccinated in Belize, Unweighted (September–October 2021)

	Predictors of vaccine uptake		Predictors of intentions to get vaccinated	
	Odds ratio	95% CI	Odds ratio	95% CI
Female	0.83	[0.56,1.23]	7.25*	[1.50,35.01]
Age groups				
Adolescents and Young Adults (15–24 years)	ref.		ref.	
Adults (25–44)	1.71	[0.95,3.08]	0.12*	[0.02,0.74]
Middle aged (45–64)	1.91	[0.85,4.30]	0.25	[0.03,1.97]
Aged (65+)	2.87*	[1.26,6.55]	1.62	[0.10,26.07]
Poorest 40%	0.66	[0.42,1.04]	1.08	[0.25,4.66]
Ethnicity				
Creole	ref.		ref.	
Garifuna	0.39	[0.11,1.38]	29.15*	[1.67,507.85]
Mixed, Others, DK	0.49	[0.18,1.30]	0.2	[0.00,11.15]
Mayan	1.67	[0.64,4.33]	8.53	[0.17,438.17]
Latino	1.29	[0.82,2.05]	4.46	[0.74,26.93]
Health conditions	1.26	[0.74,2.14]	0.37	[0.07,2.08]
District				
Belize	ref.		ref.	
Cayo	1.53	[0.89,2.65]	0.09*	[0.01,0.96]
Corozal	1.72	[0.69,4.34]	0.23	[0.02,3.28]
Orange Walk	1.08	[0.42,2.78]	0.21	[0.01,3.05]
Stann Creek	3.96**	[1.72,9.13]	0.11	[0.01,2.04]
Toledo	2.35	[0.94,5.89]	0.19	[0.00,10.96]
You will get COVID–19 and infect someone else				
Very Unlikely / Unlikely	ref.		ref.	
Likely / Very Likely / Already happened	0.99	[0.61,1.59]	0.76	[0.14,4.09]
Does not know	0.71	[0.24,2.07]	2.1	[0.10,43.49]
A friend, family member or coworker will get COVID–19 and die				
Very Unlikely / Unlikely	ref.		ref.	
Likely / Very Likely / Already happened	1.1	[0.63,1.93]	0.33	[0.05,2.16]
Does not know	1.41	[0.81,2.47]	6.09	[0.70,53.26]
Do you think most of your friends and family will get a COVID–19 vaccine?				
Some / None of them	ref.		ref.	
Most / All of them	1.39	[0.90,2.14]	3.05	[0.57,16.18]
Does not know	3.15**	[1.48,6.69]	0.72	[0.06,8.24]
Do you think most of the people in your community will get a COVID–19 vaccine?				
Some / None of them	ref.		ref.	
Most / All of them	1.47	[0.90,2.38]	0.94	[0.18,4.94]
Does not know	0.55*	[0.30,0.98]	0.54	[0.08,3.47]

What would/ has motivated you to get the COVID–19 vaccine?				
Protect my health	2.38**	[1.44,3.95]	253.04***	[24.60,2603.20]
Look for employment, work or go back to school	2.03*	[1.15,3.59]	67.75***	[7.54,609.02]
Social activities, traveling, government mandate or encouraged by others	1.52	[0.74,3.14]	83.23***	[9.11,760.11]
Nothing, freedom or no option	0.51	[0.15,1.65]	0.27	[0.01,8.87]
How safe do you think a COVID-19 vaccine is for you?				
Moderately / Very Safe	2.45**	[1.44,4.16]	2.11	[0.26,17.10]
How much protection do you think a COVID-19 vaccine would give you from getting ill-?				
Protect Moderately / A lot	2.03*	[1.12,3.66]	40.33**	[3.12,521.37]
My parents and/or sibilings think that I should get vaccinated against COVID–19				
Strongly / Somewhat disagree / Neither	ref.		ref.	
Somewhat / Strongly agree	2.07*	[1.04,4.14]	0.82	[0.06,10.56]
Does not know	2.68	[0.62,11.50]	1.15	[0.01,94.08]
People in my community think that everyone should get vaccinated against COVID–19				
Strongly / Somewhat disagree / Neither	ref.		ref.	
Somewhat / Strongly agree	0.88	[0.54,1.43]	2.45	[0.50,11.94]
Does not know	0.5	[0.25,1.02]	10.98*	[1.01,119.90]
Was it/ will it be easy/somewhat easy to get a COVID–19 vaccine?	26.92***	[13.20,54.88]	2.48	[0.65,9.50]
You will get COVID–19				
Very Unlikely / Unlikely	ref.		ref.	
Likely / Very Likely / Already happened	1.27	[0.75,2.14]	1.63	[0.32,8.35]
Does not know	1.16	[0.55,2.42]	0.09	[0.01,1.06]
A friend, family member or coworker will likely get COVID–19				
Very Unlikely / Unlikely	ref.		ref.	
Likely / Very Likely / Already happened	0.87	[0.43,1.73]	3.3	[0.42,26.15]
Does not know	1.08	[0.44,2.67]	0.1	[0.01,2.04]
You will likely have to go to a hospital if you get COVID–19				
Very Unlikely / Unlikely	ref.		ref.	
Likely / Very Likely / Already happened	0.63	[0.39,1.01]	2.17	[0.44,10.70]
Does not know	0.95	[0.50,1.79]	1.8	[0.14,23.02]
You will likely need to quarentine even if you don't get COVID–19				
Very Unlikely / Unlikely	ref.		ref.	
Likely / Very Likely / Already happened	1.49	[0.84,2.62]	0.53	[0.08,3.31]
Does not know	0.69	[0.18,2.64]	0.3	[0.01,12.88]
Protect health of others	1.05	[0.66,1.69]	1.66	[0.26,10.58]
Doctors and nurses think that everyone who has no medical problems should get vaccinated against COVID–19				
Strongly / Somewhat disagree / Neither	ref.		ref.	
Somewhat / Strongly agree	0.81	[0.43,1.52]	0.32	[0.05,1.85]
Does not know	0.93	[0.36,2.39]	0.06*	[0.01,0.69]
My close friends think that I should get vaccinated against COVID–19				
Strongly / Somewhat disagree / Neither	ref.		ref.	
Somewhat / Strongly agree	0.79	[0.39,1.60]	1.93	[0.16,23.95]
Does not know	1.58	[0.44,5.62]	0.32	[0.00,52.14]
Constant	0.01***	[0.00,0.05]	0.01*	[0.00,0.57]
N	1261		261	

* p<0.05, ** p<0.01, *** p<0.001
